# Agreement Between the Harmonized and the Self‐Explanatory Versions of the Revised ALS Functional Rating Scale in a Clinical Setting

**DOI:** 10.1002/mus.70092

**Published:** 2025-12-02

**Authors:** André Maier, Yasmin Koc, Laura Steinfurth, Dagmar Kettemann, Jenny Norden, Alessio Riitano, Phillip Schmitt, Felix Kolzarek, Senthil Subramanian, Christoph Münch, Susanne Spittel, Thomas Meyer

**Affiliations:** ^1^ Department of Neurology, Center for ALS and Other Motor Neuron Disorders Charité – Universitätsmedizin Berlin, Corporate Member of Freie Universität Berlin and Humboldt‐Universität zu Berlin Berlin Germany; ^2^ Ambulanzpartner Soziotechnologie APST GmbH Berlin Germany

**Keywords:** ALSFRS‐R, ALSFRS‐R‐SE, amyotrophic lateral sclerosis, remote digital assessment, self‐explanatory version

## Abstract

**Introduction/Aims:**

The harmonized version of the ALS Functional Rating Scale ‐ Revised (ALSFRS‐R) is typically administered according to standard operating procedures (SOPs) to ensure procedural consistency. In contrast, obtaining the self‐explanatory (SE) version of the ALSFRS‐R does not include the use of SOPs. The aim of this study was to examine the level of agreement between the harmonized and the SE version of the ALSFRS‐R in a cohort of ALS patients.

**Methods:**

In a prospective study, the harmonized ALSFRS‐R was assessed in 107 ALS patients. In parallel, all patients independently completed the ALSFRS‐R‐SE, either on a printed form (*n* = 36) or remotely via the ALS App (*n* = 71). Agreement between methods was investigated using Spearman's correlation, Lin's concordance correlation coefficient (CCC), Deming regression, Bland–Altman plots and item‐level statistics including Kendall's tau‐b and the Stuart–Maxwell test.

**Results:**

Total scores from ALSFRS‐R and ALSFRS‐R‐SE showed high correlation (*ρ* = 0.91–0.95) and concordance (CCC > 0.9). Deming regression (intercept≈0; slope≈1) and Bland–Altman analysis (95% of values within limits of agreement [LoA]) revealed no systematic bias. Item‐level agreement was high (76.6% on average), with some variability in items such as handwriting, walking, and dyspnea. ALS progression rates were consistent (differences ≤ 0.02). ALSFRS‐R‐SE remained robust across remote digital and paper‐based assessments.

**Discussion:**

The strong agreement between the harmonized and self‐explanatory versions of the ALSFRS‐R supports their interchangeable use. The SE format may facilitate remote digital assessment and reduce complexity of ALSFRS‐R assessment in research and clinical practice. Further studies are warranted to validate the ALSFRS‐R‐SE across larger cohorts, multiple languages, and diverse rater groups.

**Abbreviations**
ALS: amyotrophic lateral sclerosis
ALSFRS‐R: ALS Functional Rating Scale ‐ Revised
ALSFRS‐R‐SE: ALS Functional Rating Scale ‐ Revised ‐ Self‐Explanatory
CCC: concordance correlation coefficient
CoV: coefficient of variation
HCP: healthcare professional
LoA: limits of agreement
PR: progression rate
SE: ALSFRS‐R‐SE
SOP: standard operating procedure

## 1 Introduction

In amyotrophic lateral sclerosis (ALS), the ALS Functional Rating Scale ‐ Revised (ALSFRS‐R) is an established instrument to record motor function and ALS‐related symptoms in both research and clinical care [1, 2]. The scale is most commonly administered through an interview conducted by a healthcare professional (HCP), with the interviewer undergoing training to ensure data is collected in as standardized a manner as possible. The standard operating procedures (SOPs) for conducting the interviews have undergone a harmonization process [3]. Despite the implementation of a harmonized SOP aimed at minimizing inter‐ and intra‐rater variability, fundamental limitations of the standardized ALSFRS‐R assessment remain. Thus, the change of evaluators was repeatedly identified as a key factor in sudden changes in the assessment of functional deficits [4, 5]. To overcome the recurring issues of changing evaluators, patient self‐assessment has been proposed, leading to the development of several adapted forms of the ALSFRS‐R for self‐administration [6, 7, 8, 9]. Moreover, a self‐explanatory version of the ALSFRS‐R (ALSFRS‐R‐SE) was developed and formally agreed upon through a consensus process by an ALS expert group and has been published in English and German [10]. This version provides additional, clearly understandable explanations and specifications for each item. Beyond patient self‐rating, the ALSFRS‐R‐SE is aimed to be used by HCPs, even without training. The ALSFRS‐R‐SE contributes to the increasing trend toward digitalization and the use of personal smartphones for ALSFRS‐R assessment [11]. Recently, when the ALSFRS‐R‐SE was used for remote digital assessment via smartphone, the intrasubject variability, an important confounder in trial settings, was demonstrated to be non‐inferior compared to clinical capture [12].

Despite the broader use of the ALSFRS‐R‐SE in German‐speaking countries, uncertainties persist regarding how the harmonized and self‐explanatory versions of the ALSFRS‐R may affect the variability [13]. To address these uncertainties, the aim of this study was to compare the results of the harmonized and the self‐explanatory ALSFRS‐R in a cohort of ALS patients.

## 2 Methods

### 2.1 Study Design

This study was a prospective longitudinal single‐center study. Conducting and reporting the study was guided by the COnsensus‐based Standards for the selection of health Measurement INstruments (COSMIN) [14], although only part of the complex framework was applied because all of the measurement properties of the instruments have not been studied. The investigation took place between September 2023 and February 2025.

### 2.2 Variables

#### 2.2.1 ALSFRS‐R

The ALSFRS‐R is a disease‐specific, validated instrument for assessing motor impairment in four domains: bulbar, fine motor, gross motor, and respiratory function. It comprises 12 items with 5 scoring options for each item (0–4). The total range of the scale is from 0 (no function) to 48 scale points (full function). The four domains are typically analyzed as subscales, as they allow a higher degree of differentiation compared to the total score [15], which reflects the overall disease severity.

#### 2.2.2 Harmonized Standard Operation Procedure of Administering the ALSFRS‐R

The ALSFRS‐R and its predecessor, the ALSFRS, were originally designed as a survey instrument for clinical trials, in which HCPs could conduct a structured interview with patients about their symptoms and then transcribe the answers into the most appropriate form for the scale items [1, 16]. The trainings that then emerged, first in the USA through the Northeast ALS Consortium (NEALS) and then in Europe through the Treatment Research Initiative to Cure ALS (TRICALS), pursued the purpose of ensuring that the assessment was as similar as possible in order to reduce intra‐rater and the far more significant inter‐rater variability [5]. However, the harmonization between the two entities has resulted in an SOP that addresses many of the uncertainties of the original scale and provides some important considerations regarding possible patient responses. With the new SOP, both NEALS and TRICALS are committed to providing training in a consistent manner [3].

#### 2.2.3 ALSFRS‐R‐SE

The ALSFRS‐R‐SE was developed in a consensus initiative by a group of German ALS clinicians and researchers [10]. All participants in the process had extensive experience with the administration and evaluation of the ALSFRS‐R. The motivation for this activity was the awareness of inconsistent SOPs, the lack of a uniform German version of the ALSFRS‐R at the time, and the desire to resolve different scales in different settings. The premise of the ALSFRS‐R‐SE was always to reflect the original scale as closely as possible, without altering it. By providing explanatory notes, easy language, and a clear introductory text, all persons, patients and HCPs alike, would be able to perform ALS assessments without training. This makes the scale a valuable instrument in a clinical and trial environment, where, in addition to traditional assessment situations, the self‐evaluation of patients using remote digital methods, such as the ALS App, is becoming increasingly important [12].

### 2.3 Procedure

#### 2.3.1 Assessment of Harmonized ALSFRS‐R and ALSRS‐R‐SE

The comparison between the harmonized ALSFRS‐R and the SE was conducted on the same patient using the most common method, where the German version of each scale was used, even though training on the harmonized ALSFRS‐R and its SOP was usually provided in English. Therefore, the interview was conducted by HCPs and the ALSFRS‐R‐SE (SE) assessment was performed by the patients themselves. Two experienced ALS study nurses, trained and certified in the harmonized SOP but without prior experience with the SE, conducted the interviews during two consecutive visits in the ALS department. For the SE assessment, independent of the interview, the patient was asked to complete either the paper version or the remote digital version using the ALS App, compatible with iOS and Android devices (https://www.ambulanzpartner.de/als‐app/). If patients stated that they had completed the SE on their smartphone a maximum of 3 days before the visit or that they intended to complete it at home up to 3 days afterwards, this data was used, without a predefined minimum interval between the two assessments.

#### 2.3.2 Patients and Sample Size

The statistical method of choice for presenting the agreement of an assessment instrument that was either collected by different raters or using different methods is to determine the level of concordance using the Bland and Altman method with LoA [17], which allows for a nuanced visualization and quantification of systematic bias and variability between measurements. That formed the basis for the selection of the method of sample size estimation [18]. In order to calculate the sample size, we used values from preliminary studies that already reported distances of LoA and standard deviations of the differences [7, 8]. In accordance with these studies, the standard deviation of the differences was set to 2.5, and LoA < 7 points were considered acceptable. A value of 0 was chosen for the bias, as the literature reports only minor systematic deviations. Given a significance level of 0.05 and a power of 80%, a total case number of at least 30 subjects per cohort was recommended. Since remote digital assessment was preferred, recruitment at ALS outpatient clinic at Charité continued until the print cohort had reached the projected number.

### 2.4 Protocol Approvals and Registrations

The study protocol was approved by the Medical Ethics Committee of Charité – Universitätsmedizin Berlin, Germany (EA2/190/23). Written informed consent was obtained from all participants.

### 2.5 Statistical Methods

Descriptive statistics were used for the statistical analysis (mean, standard deviation in ± and ranges). The selection of methods for comparing the instruments, the harmonized ALSFRS‐R interview and ALSFRS‐R‐SE, followed the Recommendations of COSMIN and the ISPOR ePRO Task Force [19]. As an interview‐based questionnaire, it was compared with a paper‐based and an electronic patient‐reported outcome (ePRO) that represents a further development of the questionnaire. To assess the relationship and agreement between the two versions of the questionnaire in two cohorts, multiple statistical approaches were applied. Spearman's rank correlation coefficient (*ρ*) was used to evaluate the monotonic association between the total scores of the ALSFRS‐R interview and ALSFRS‐R‐SE. In addition, Lin's concordance correlation coefficient (CCC) was calculated to quantify the concordance by accounting for both precision and accuracy [20]. To further examine potential systematic differences and proportional bias between the two score sets, a Deming regression was conducted [21]. This regression method accounts for measurement error in both variables by estimating a line of best fit that minimizes the orthogonal distances between the observed data points and the regression line. The resulting intercept and slope parameters were interpreted to assess fixed and proportional bias, respectively. Additionally, a Bland–Altman analysis was performed to visualize the agreement between the two measurement methods and to identify any systematic bias [17]. Mean differences and 95% LoA (mean ± 1.96 SD) were calculated and plotted against the mean of the two measurements. Additionally, progression rates (PRs) were calculated as the monthly decline of the ALSFRS‐R and ‐SE total score [(48 − ALSFRS‐R/‐SE)/disease duration in months], since changes of PR are established study outcomes and potential differences between cohorts may affect comparative analyses [12]. PR was categorized into slow, medium, and fast groups [22]. To evaluate the agreement and consistency of individual item responses, the following statistical methods were employed. Mean differences between item scores were calculated to assess potential systematic shifts in response tendencies across modalities. Kendall's Tau‐b (τ) was used to evaluate the ordinal association between item responses, providing a nonparametric measure of rank correlation that accounts for ties [23]. This was particularly used given the ordinal nature of the item response scales. To assess marginal homogeneity across paired categorical responses, the Stuart–Maxwell test was applied [24]. This test examines whether the distribution of item responses differs significantly between the two administration modes, allowing the detection of systematic shifts in response patterns at the item level. All analyses were conducted using R version 4.4.2 (2024‐10‐31), and statistical significance was set at *p* < 0.05.

## 3 Results

### 3.1 Demographic and Clinical Characteristics

**FIGURE 1:**
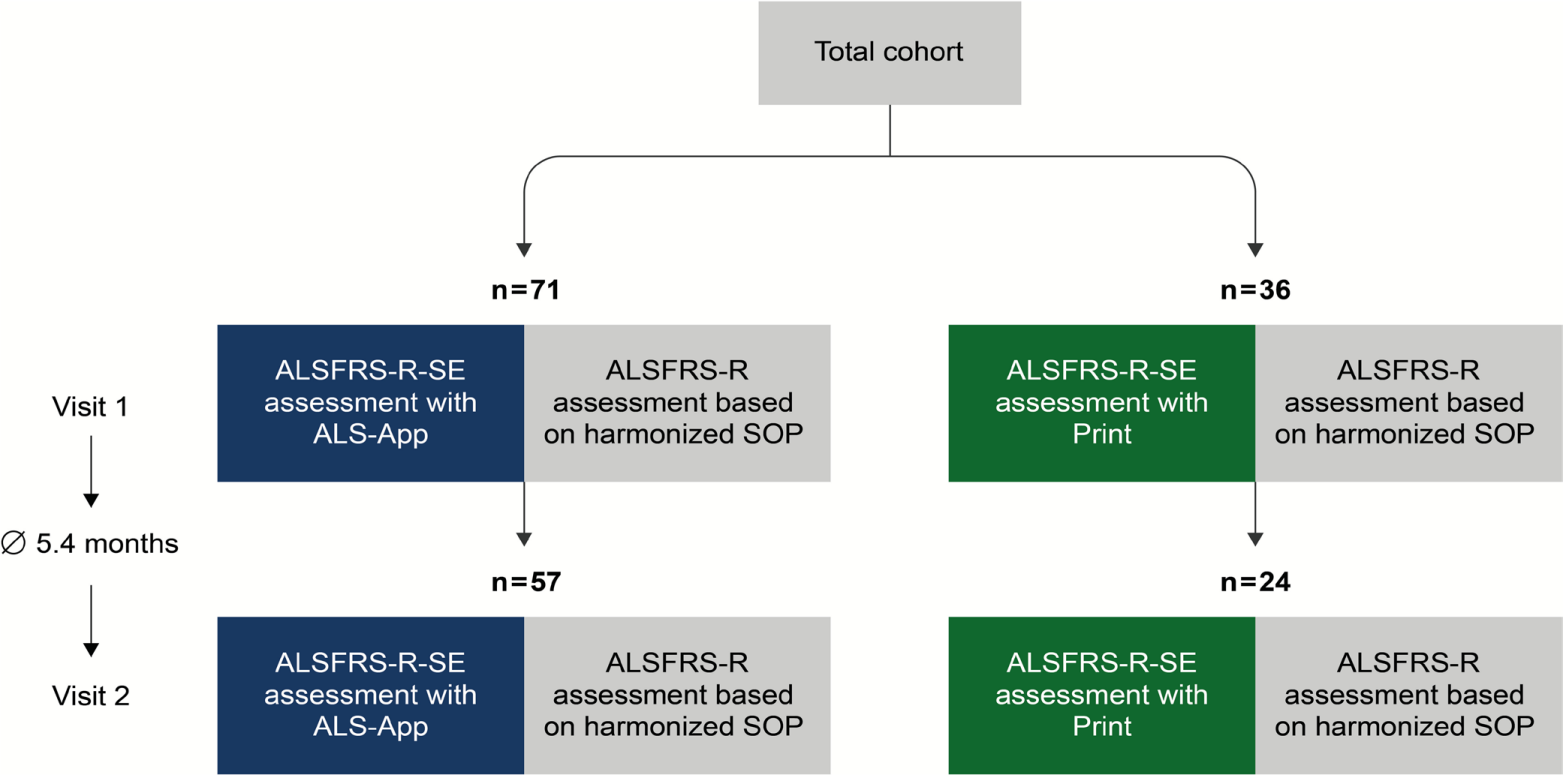
Flowchart of the patient cohort. Participants were assigned to one of two groups depending on the mode of ALSFRS‐R‐SE collection: Remote digital assessment via the ALS App or completion on a printed form. At two study visits, the ALSFRS‐R was administered through an interview based on the harmonized SOP.

This study included 107 ALS patients who provided at least one ALSFRS‐R‐SE (SE) data set, either on the App or on a printed form, and a harmonized ALSFRS‐R interview. Follow‐up visits were carried out on 81 patients after 5.4 months on average (SD 55.5) of the initial visit (Figure 1).

The demographics and clinical characteristics of the participants are presented in Table 1. There were no differences between the ALS App cohort and the print cohort regarding sex distribution, ALSFRS‐R, or SE total scores. Participants who completed the ALSFRS‐R‐SE on paper were significantly older, had a longer disease duration, and showed a slower PR.

**TABLE 1:** Demographic and clinical characteristics of participants at baseline, *n* = 107.

Characteristics	Classification	Total cohort	ALS App cohort	Print cohort	*p* ^*a*^
Sex	Female, % (n)	39.4 (43)	39.4 (28)	41.7 (15)	0.824
	Male, % (n)	60.6 (64)	60.6 (43)	58.3 (21)	
Age	At onset, years, mean (SD, R)	58.4 (10.6, 25.4–83.9)	57.7 (10.2; 25.4–83.9)	59.8 (11.3; 30.6–81.5)	0.272
	At visit 1, years, mean (SD, R)	63.2 (9.4, 41.7–87.2)	61.2 (8.8; 41.7–84.9)	67.2 (9.4; 44.2–87.2)	0.001
Disease duration	At visit 1, months, median (IQR)	29.77 (15.9–55.5)	28.9 (14–45)	40.3 (20.6–141.1)	0.003
ALSFRS‐R‐SE total score (maximum 48)	Mean (SD, R)	35.1 (8.3, 10–47)	35.6 (8.1; 9–47)	33.4 (8.5; 11–46)	0.059
ALSFRS‐R interview (maximum 48)	Mean (SD, R)	34.9 (8.3, 9–47)	36.1 (8.1; 12–47)	33.1 (8.4; 10–46)	0.156
Rate of disease progression	Mean (SD, R)	0.50 (0.54, 0.01, 3.70)	0.57 (0.4; 0.01–3.70)	0.37 (0.04–2.79)	0.049

Abbreviations: ALSFRS‐R, Amyotrophic Lateral Sclerosis Functional Rating Scale—Revised; ALSFRS‐R‐SE, Amyotrophic Lateral Sclerosis Functional Rating Scale—Revised—Self‐Explanatory; IQR, interquartile range; *n*, number of participants; R, range; SD, standard deviation.

^a^ Pearson's *χ* ^2^ test; Wilcoxon rank sum test.

### 3.2 Comparison of Harmonized ALSFRS‐R and ALSFRS‐R‐SE Total Scores

#### 3.2.1 Correlation, Concordance and Regression of Harmonized ALSFRS‐R and ALSFRS‐R‐SE Total Scores

**FIGURE 2:**
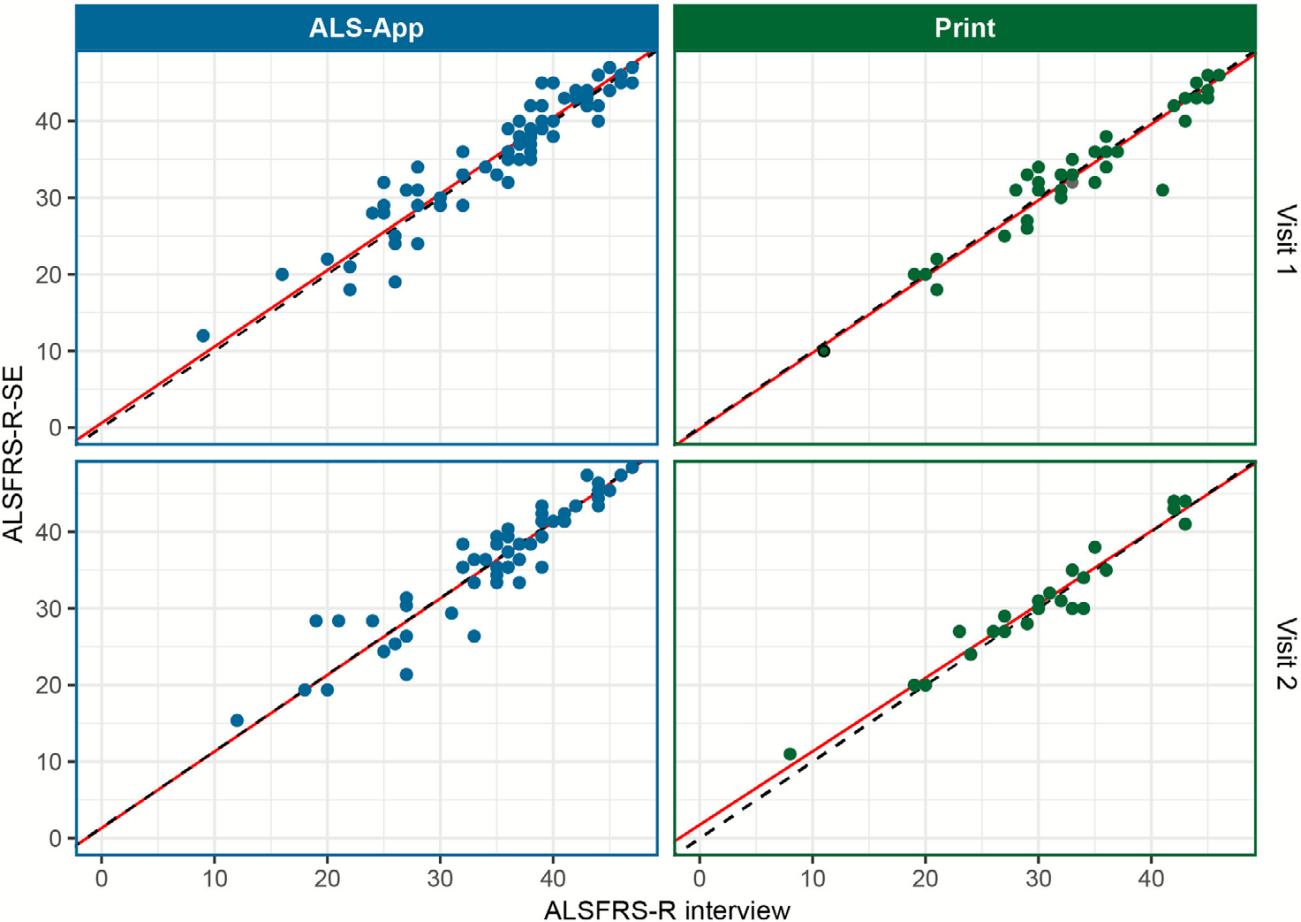
Correlation and deming regression of harmonized ALSFRS‐R interview and ALSFRS‐R‐SE total scores across the two cohorts of ALS App and paper based ALSFRS‐R‐SE assessment and at two time points (visits 1 and 2). The correlation of the total scores is shown by the proximity to the bisecting line (dashed line). Deming regression is represented by the deviation (intercept and slope) of the bisecting line and the estimated line of best fit (red line).

At all visits, both in the ALS App and in the print cohort, there was a high Spearman's correlation and concordance (CCC) between the total values with coefficients above 0.9 (Table 2). The Deming regression revealed no statistically significant difference from the estimated line of best fit, as no fixed or proportional bias was found, neither at the individual nor at the aggregated time points (Figure 2; Table 2).

**TABLE 2:** Correlation, concordance, and regression of total scores between the harmonized ALSFRS‐R SOP, which was recorded as an interview, and the ALSFRS‐R SE, which was completed by the patient at two time points.

			Correlation		Concordance		Deming regression					
Cohort	Visit	*N*	Spearman's *ρ*	*p*	CCC	95% CI	Intercept	95% CI	*p*	Slope	95% CI	*p*
App	1	71	0.95	< 0.001	0.97	0.91; 0.97	0.6	−3.17; 4.37	0.76	1	0.9; 1.09	0.95
App	2	57	0.92	< 0.001	0.94	0.88; 0.96	−0.04	−4.8; 4.71	0.99	1	0.88; 1.12	0.98
Print	1	36	0.91	< 0.001	0.98	0.91; 0.98	−0.16	−2.52; 2.19	0.89	0.99	0.93; 1.06	0.86
Print	2	24	0.95	< 0.001	0.97	0.93; 0.99	1.77	−1.39; 4.93	0.26	0.96	0.86; 1.06	0.41

Abbreviation: CCC, concordance correlation coefficient.

#### 3.2.2 Systemic Error and Agreement of Harmonized ALSFRS‐R and ALSFRS‐R‐SE Total Scores

The systematic error is represented by the mean difference between the mean total scores. There was no statistically significant difference or specific deviation in one direction. Patients in the largest group, who rated themselves via the ALS App at visit 1, tended to rate themselves slightly higher. However, this was not the case for visit 2 or in the print cohort visit 1, but again in the smallest group of the print cohort at visit 2 (Table 3). The coefficients of variation (CoVs), that is, the relative standard deviation, are also nearly equal, which reflects a comparable distribution in the two versions of ALSFRS‐R.

**TABLE 3:** Systemic error and agreement of total scores between the harmonized ALSFRS‐R SOP, which was recorded as an interview, and the ALSFRS‐R SE, which was completed by the patient at two time points.

			Harmonized ALSFRS‐R		ALSFRS‐R‐SE		Difference		Limits of agreement		
Cohort	Visit	*N*	Mean (SD)	CoV	Mean (SD)	CoV	Mean (SD)	*p* ^a^	Lower limit	Upper limit	95% CI
ALS App	1	71	35.63 (8.12)	0.23	36.13 (8.09)	0.22	−0.49 (2.64)	0.11	−5.67	4.68	−6.66; 5.67
ALS App	2	57	34.79 (7.58)	0.22	34.7 (7.57)	0.22	0.09 (2.83)	1	−5.46	5.63	−6.66; 6.84
Print	1	36	33.44 (8.47)	0.25	33.08 (8.42)	0.25	0.36 (2.5)	0.47	−4.53	5.26	−4.53; 6.65
Print	2	24	30.58 (8.26)	0.27	31.08 (7.92)	0.27	−0.5 (1.89)	0.18	−4.2	3.2	−5.56; 4.56

Abbreviation: CoV, coefficient of variation.

^a^ Wilcoxon rank sum test.

**FIGURE 3:**
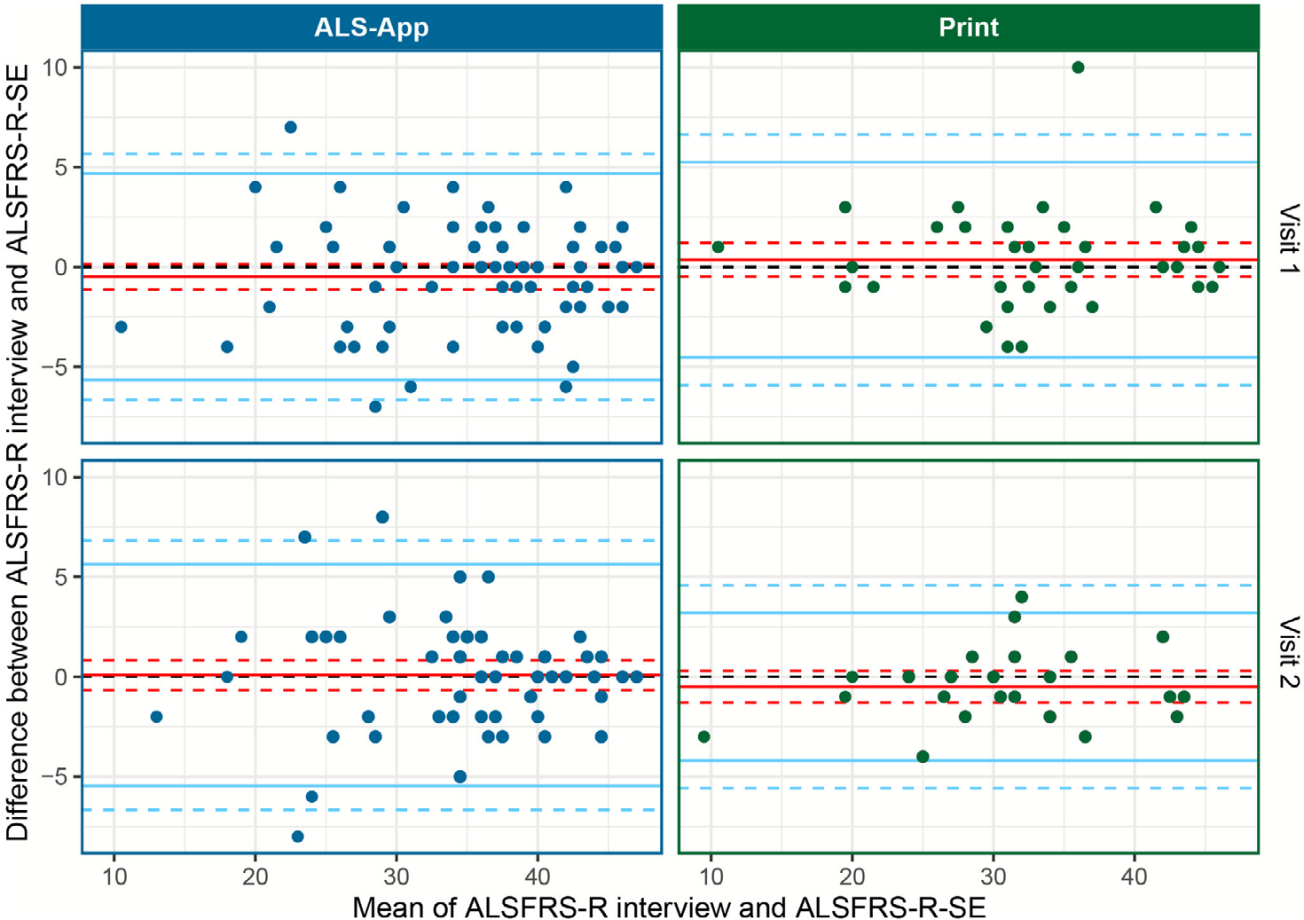
Bland–Altman plot of harmonized ALSFRS‐R interview and ALSFRS‐R‐SE total scores across the two cohorts of App and paper based ALSFRS‐R‐SE assessment and at two time points (visits 1 and 2). The line of mean differences (bias) is shown as a continuous red line, while a dashed red line indicates the 95% confidence intervals. The limits of agreement are shown as a continuous blue line, with a dashed blue line representing its 95% confidence interval.

The agreement between the harmonized ALSFRS‐R and ALSFRS‐R‐SE was very high in both cohorts and at both timepoints (Figure 3, Table 3). More than 95% of all pairs of measurements were within the LoA. Except for visit 2 in the print cohort, where the LoA were narrower, they were comparable across all conditions.

### 3.3 Single‐Item Analysis of Harmonized ALSFRS‐R and ALSFRS‐R‐SE

The absolute difference between the individual items is on average only 0.12 (SD 0.1; range −0.25 to 0.21) (Table).

**FIGURE 4:**
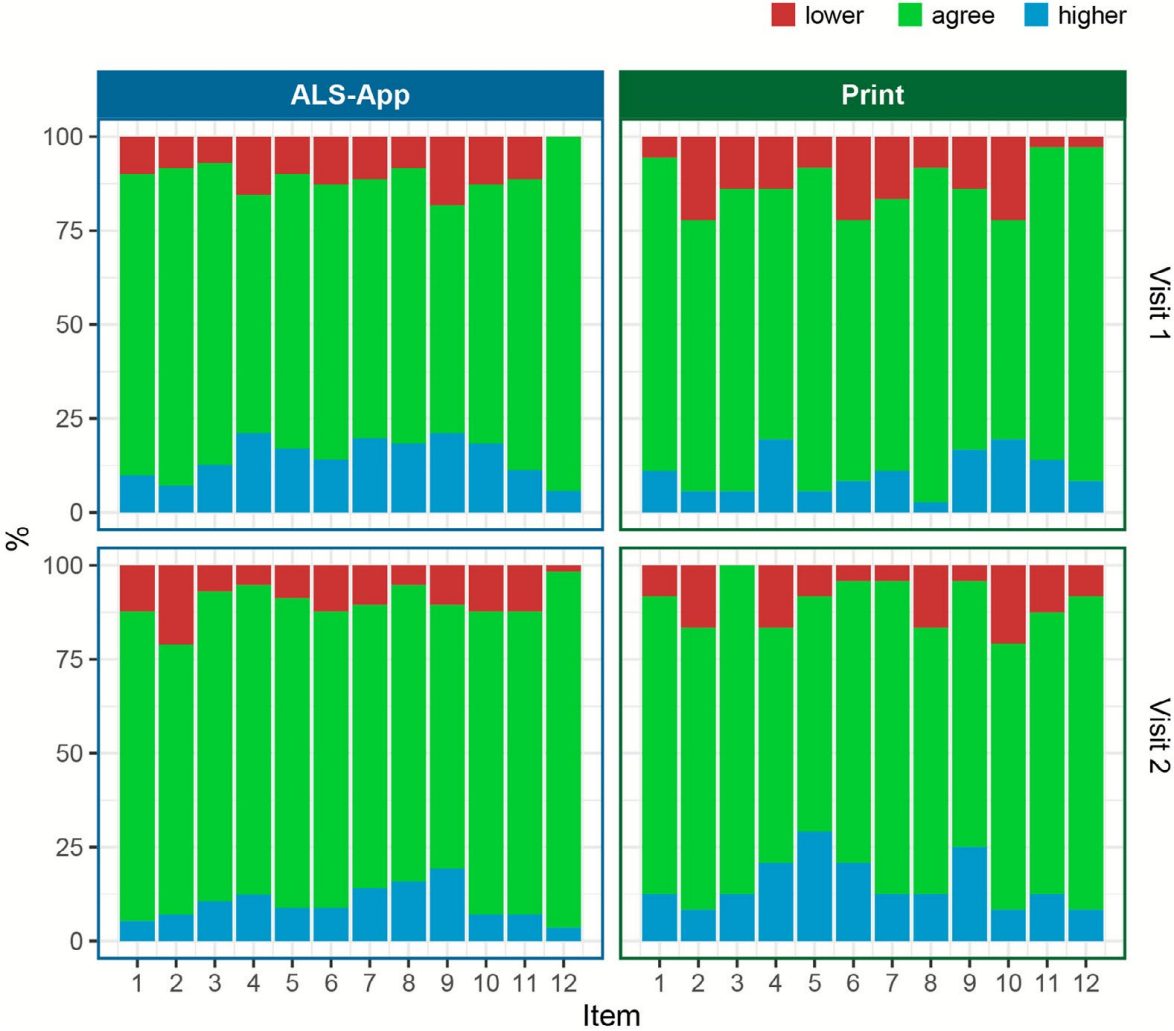
Proportion of agreement in percent and deviation of the singe items of harmonized ALSFRS‐R interview and ALSFRS‐R‐SE. The agreement between the measurements is represented by green color, the deviation upwards by red and downwards by blue.

The study revealed that although the agreement of most items was high with 76.6% on average across all items, certain items showed only moderate agreement values in individual visits (Table 4). Uncertainty intervals that reached below 50% were observed for item 4 (handwriting; in the print cohort at visits 1 and 2), item 5 (cutting food/handling utensils; in the print cohort at visit 2), item 8 (walking; in the print cohort at visit 2), item 9 (climbing stairs; in the app cohort at visit 1 and print cohort at visit 2), and item 10 (dyspnea, in the print cohort at visits 1 and 2). However, it is noticeable that the rank correlation, as assessed by Kendall's *τ* coefficient, remained high for these items. In contrast, some items exhibited low rank correlations (e.g., item 11 in the app cohort at visit 1, or item 10 in the print cohort at visit 2), resulting in good percent agreement. This occurs when a large number of measurement pairs are identical; however, if there are deviations, those are nonsystematic (Figure S1). In accordance with this, the Stuart–Maxwell test consistently showed that there were no systematic deviations in these pairs, as in all others. These nonsystematic deviations can also be seen graphically (Figure 4), with an average percentage of disagreement being 11.7% (SD 6.0, range 0.0–29.2) in either direction (Table S1). However, no item in particular stands out as exhibiting particularly low levels of agreement across both cohorts and visits.

**TABLE 4:** Agreement and correlation of individual ALSFRS‐R items between the harmonized ALSFRS‐R SOP recorded as an interview and the ALSFRS‐R‐SE completed by the patient at two time points. For empty table cells, the Stuart–Maxwell test could not be applied due to low variability.

Item	ALS App								Print							
	Visit 1				Visit 2				Visit 1				Visit 2			
	Percent agreement (95% CI)	Kendalls *τ*	Spearman	*p* ^a^	Percent agreement (95% CI)	Kendalls *τ*	Spearman	*p* ^a^	Percent agreement (95% CI)	Kendalls *τ*	Spearman	*p* ^a^	Percent agreement (95% CI)	Kendalls *τ*	Spearman	*p* ^a^
1	80.3 (69.1; 88.8)	0.84*	0.87*	0.62	82.5 (70.1; 91.3)	0.89*	0.93*	0.26	83.3 (67.2; 93.6)	0.88*	0.92*	0.86	79.2 (57.8; 92.9)	0.84*	0.88*	0.99
2	84.5 (74.0; 92.0)	0.9*	0.93*	0.27	71.9 (58.5; 83.0)	0.7*	0.73*	0.11	72.2 (54.8; 85.8)	0.75*	0.82*	0.33	75 (53.3; 90.2)	0.82*	0.86*	0.5
3	80.3 (69.1; 88.8)	0.83*	0.85*	0.27	82.5 (70.1; 91.3)	0.85*	0.88*	0.23	80.6 (64.0; 91.8)	0.79*	0.82*	—	87.5 (67.6; 97.3)	0.92*	0.94*	0.56
4	63.4 (51.1; 74.5)	0.75*	0.82*	0.73	82.5 (70.1; 91.3)	0.85*	0.9*	0.15	66.7 (49.0; 81.4)	0.79*	0.85*	0.83	62.5 (40.6; 81.2)	0.78*	0.85*	0.5
5	73.2 (61.4; 83.1)	0.79*	0.85*	0.72	82.5 (70.1; 91.3)	0.84*	0.87*	0.74	86.1 (70.5; 95.3)	0.92*	0.95*	—	62.5 (40.6; 81.2)	0.83*	0.9*	—
6	73.2 (61.4; 83.1)	0.85*	0.9*	0.07	78.9 (66.1; 88.6)	0.83*	0.87*	0.55	69.4 (51.9; 83.7)	0.82*	0.88*	0.4	75 (53.3; 90.2)	0.87*	0.92*	0.41
7	69 (56.9; 79.5)	0.81*	0.87*	0.41	75.4 (62.2; 85.9)	0.82*	0.87*	0.86	72.2 (54.8; 85.8)	0.83*	0.9*	0.47	83.3 (62.6; 95.3)	0.88*	0.92*	0.41
8	73.2 (61.4; 83.1)	0.81*	0.86*	0.46	78.9 (66.1; 88.6)	0.88*	0.91*	0.07	88.9 (73.9; 96.9)	0.91*	0.93*	0.74	70.8 (48.9; 87.4)	0.83*	0.89*	0.68
9	60.6 (48.3; 72.0)	0.73*	0.81*	0.55	70.2 (56.6; 81.6)	0.84*	0.9*	0.771	69.4 (51.9; 83.7)	0.87*	0.92*	—	70.8 (48.9; 87.4)	0.88*	0.92*	0.29
10	69 (56.9; 79.5)	0.74*	0.82*	0.24	80.7 (68.1; 90.0)	0.73*	0.78*	0.11	58.3 (40.8; 74.5)	0.54*	0.59*	0.26	70.8 (48.9; 87.4)	0.32*	0.33*	0.68
11	77.5 (66.0; 86.5)	0.41*	0.43*	0.81	80.7 (68.1; 90.0)	0.64*	0.69*	0.98	83.3 (67.2; 93.6)	0.76*	0.78*	0.45	75 (53.3; 90.2)	0.79*	0.84*	0.51
12	94.4 (86.2; 98.4)	0.98*	1*	0.47	94.7 (85.4; 98.9)	0.93*	0.95*	0.56	88.9 (73.9; 96.9)	0.65*	0.67*	0.74	83.3 (62.6; 95.3)	0.7*	0.74*	0.98

^a^ Stuart–Maxwell test.

* *p* < 0.001.

### 3.4 Comparison of PRs

The PR across individual cohorts and visits, as well as the distribution of patients into PR categories, are presented in Table S2. The categorical evaluation revealed high agreement values (Table S3) and a high correlation in all categories (Figure S2). There was no relevant or statistically significant systematic deviation between the PR of the two ALSFRS‐R versions of the harmonized SOP interview and the ALSFRS‐R‐SE.

## 4 Discussion

### 4.1 Agreement Across Total Scores and Individual Items

This study demonstrates a high degree of agreement between the conventional, interview‐administered ALSFRS‐R and the self‐administered ALSFRS‐R‐SE. Across both the paper‐based and digital format, the total scores were highly concordant, and in contrast to previous reports [9, 25], no consistent systematic bias was observed. Item‐level analyses revealed overall robust correlations, although some items showed greater variability. Taken together, these results support the clinical usability of the ALSFRS‐R‐SE as a reliable alternative to the conventional ALSFRS‐R.

### 4.2 Comparison With Previous Evidence and Clinical Practice

The ALSFRS‐R‐SE introduces a self‐explanatory concept that goes beyond conventional self‐administered approaches [10]. It is designed to be used both by patients and by HCP without the need for specific training. By providing clear explanations for each item, the SE reduces dependence on SOPs and rater training, which have been the primary means to mitigate inter‐rater variability in the use of the ALSFRS‐R [5]. Earlier studies suggested that patient self‐ratings may be systematically higher than interviewer ratings [9, 25]. Our results do not support this concern as no consistent directional bias was detected. This finding indicates that the explanatory structure of the SE may actively attenuate interpretative variability rather than reproduce it.

From a clinical perspective, the SE thus represents more than a practical alternative. It has the potential to democratize the use of ALSFRS‐R assessments and it lends itself naturally to digital implementation, as demonstrated by its integration into the ALS App as an ePRO based on a bring your own device (BYOD) model [26]. By facilitating broader dissemination, scalability, and cost‐efficiency, it addresses features that are increasingly critical not only in multicenter trials [27] but also in clinical practice and registries enabling larger datasets [28]. Recent findings indicate that most patients consider this self‐assessment relevant and not burdensome [29]. The present results are also consistent with a previous non‐inferiority study, reinforcing the robustness and reliability of the ALSFRS‐R‐SE as an alternative to interview‐based administration [12]. Beyond clinical usability, the ALSFRS‐R‐SE has been adopted as a primary functional outcome measure in a large multicenter biomarker study, linking functional trajectories with neurofilament dynamics [30].

### 4.3 Item‐Level Considerations

Although overall correlations were strong, some variability was observed for individual items, particularly for those assessing “climbing stairs” (item 9) and “dyspnea” (item 10). These items may be more difficult to assess, since patients and raters interpret them differently despite SOPs or explanatory notes and they rely on subjective experiences of exertion and respiratory function [31, 32]. Importantly, however, no consistent directional bias was present, and rank correlations remained high. This reinforces the notion that the ALSFRS‐R should not be interpreted as a total score, but rather should be analyzed in a more differentiated approach, for example, by considering the subscales [33, 34, 35].

### 4.4 Study Limitations

The primary limitations of this study are its relatively small size and single‐center design. Compared with typical trial cohorts, the study population showed slower progression, longer disease duration, and less standardized assessment intervals. These differences may have affected data robustness and limited the transferability of these findings to clinical trial settings, in which participants are typically followed at regular intervals and may exhibit larger ALSFRS‐R changes over time. Participation in the ALS App‐based assessment was optional, which reflects a realistic use case and explains the reduced number of follow‐up visits and variable intervals between ALSFRS‐R assessments.

Another limitation concerns the generalizability of the findings to other settings in which ALSFRS‐R data are collected. In this study, assessments were conducted by experienced interviewers trained according to the established SOP [3], and patients may have been familiar with the ALSFRS‐R‐SE due to its routine use in the ALS App and at the study center [12]. In addition, potential carryover bias cannot be excluded, as both assessments were completed within a certain time frame but without a predefined minimum interval. These conditions cannot be assumed in other contexts and may introduce greater variability, particularly in clinical trials settings.

### 4.5 Conclusion

The results of this study comparing the conventional, interviewer‐administered ALSFRS‐R and the self‐administered ALSFRS‐R‐SE demonstrate a high level of agreement between the two methods. The observed variability cannot be attributed to either approach, as no relevant systematic differences in score distribution were found. Item‐level analysis revealed no consistent directional bias but highlighted specific items with greater variability, emphasizing the value of further item‐focused investigation. The use of the SE across cultural and linguistic contexts should be further evaluated and expert consensus may enable harmonized application without repeated validation [36]. Given the substantial share of German‐speaking ALS patients within the European Medicines Agency (EMA) regulatory framework, this study provides essential groundwork for broader validation of the ALSFRS‐R‐SE and its potential regulatory acceptance by authorities such as the EMA and the US Food and Drug Administration (FDA). Although the study was conducted within the EMA region, the findings remain equally relevant for regulatory decision‐making in FDA‐regulated contexts.

## Author Contributions

**André Maier:** conceptualization, writing – original draft, methodology, writing – review and editing, investigation, formal analysis, data curation, project administration. **Yasmin Koc:** investigation, data curation. **Laura Steinfurth:** conceptualization, investigation, data analysis. **Dagmar Kettemann:** writing – review and editing, investigation. **Jenny Norden:** writing – review and editing, investigation. **Alessio Riitano:** investigation, data curation. **Phillip Schmitt:** investigation, data curation. **Felix Kolzarek:** investigation, data curation. **Senthil Subramanian:** writing – review and editing, data analysis. **Christoph Münch:** supervision, resources, data curation. **Susanne Spittel:** writing – review and editing, data analysis. **Thomas Meyer:** writing – review and editing, project administration, supervision, resources, data curation, conceptualization, funding acquisition.

## Funding

This work was supported by the Boris Canessa ALS Stiftung (Düsseldorf, Germany) and Martin Herrenknecht Fonds for ALS Research (H4017703513237604).

## Ethics Statement

We confirm that we have read the Journal's position on issues involved in ethical publication and affirm that this report is consistent with those guidelines.

## Conflicts of Interest

A.M. is on an advisory board of Novartis and has received consulting fees and honoraria for presentations from Zambon GmbH, ITF Pharma, and Roche. T.M. is on the advisory board of Biogen and has received consulting fees from Biogen. T.M. and C.M. are founders and shareholders of the Ambulanzpartner Soziotechnologie APST GmbH, which makes the internet platform Ambulanzpartner and the mobile application “ALS App.” The other authors declare no conflicts of interest.

## Supporting information


**Figure S1:** mus70092‐sup‐0001‐Supplement_Figure_S1.pdf.

**Figure S2:** mus70092‐sup‐0002‐Supplement_Figure_S2.pdf.

**Table S1:** mus70092‐sup‐0003‐Supplement_Table_S1.pdf.

**Table S2:** mus70092‐sup‐0004‐Supplement_Table_S2.pdf.

**Table S3:** mus70092‐sup‐0005‐Supplement_Table_S3.pdf.

## Data Availability

The data that support the findings of this study are available from the corresponding author upon reasonable request.
