# Supplementary material for: Agreement Between the Harmonized and the Self‐Explanatory Versions of the Revised ALS Functional Rating Scale in a Clinical Setting
Source: Muscle Nerve. 2025 Dec 2;73(2):250–9. doi: 10.1002/mus.70092 (PMC12803583; doi:10.1002/mus.70092)
Supplement: Supplementary file 1 — Figure S1: mus70092‐sup‐0001‐Supplement_Figure_S1.pdf. [file MUS-73-250-s001.pdf]

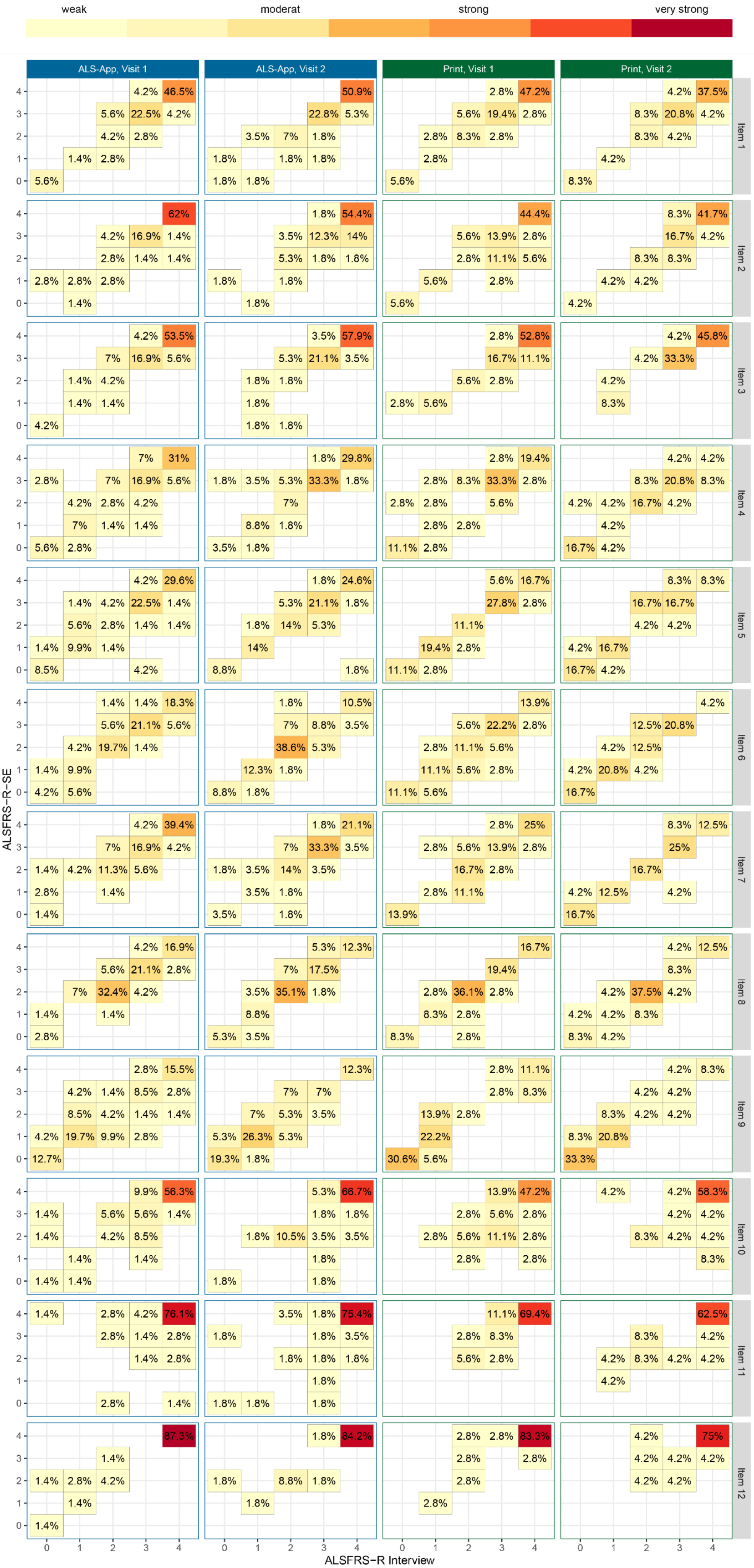
weak
moderat
strong
very strong
ALS-App, Visit 1
ALS-App, Visit 2
Print, Visit 1
Print, Visit 2
Item 1
Item 2
Item 3
Item 4
Item 5
Item 6
Item 7
Item 8
Item 9
Item 10
Item 11
Item 12
ALSFRS−R−SE
ALSFRS−R Interview

Supplement Figure S1: Single-item analysis of the comparison between harmonized ALSFRS-R interview (x-axis) and ALSFRS-R-SE (y-axis). The proportion of patients with the corresponding characteristics of the respective ALSFRS-R versions is shown in each field. A darker color indicates a higher proportion with a stronger correlation, respectively. Fields on the bisecting axis indicate an exact match.
