# Supplementary material for: Agreement Between the Harmonized and the Self‐Explanatory Versions of the Revised ALS Functional Rating Scale in a Clinical Setting
Source: Muscle Nerve. 2025 Dec 2;73(2):250–9. doi: 10.1002/mus.70092 (PMC12803583; doi:10.1002/mus.70092)
Supplement: Supplementary file 2 — Figure S2: mus70092‐sup‐0002‐Supplement_Figure_S2.pdf. [file MUS-73-250-s003.pdf]

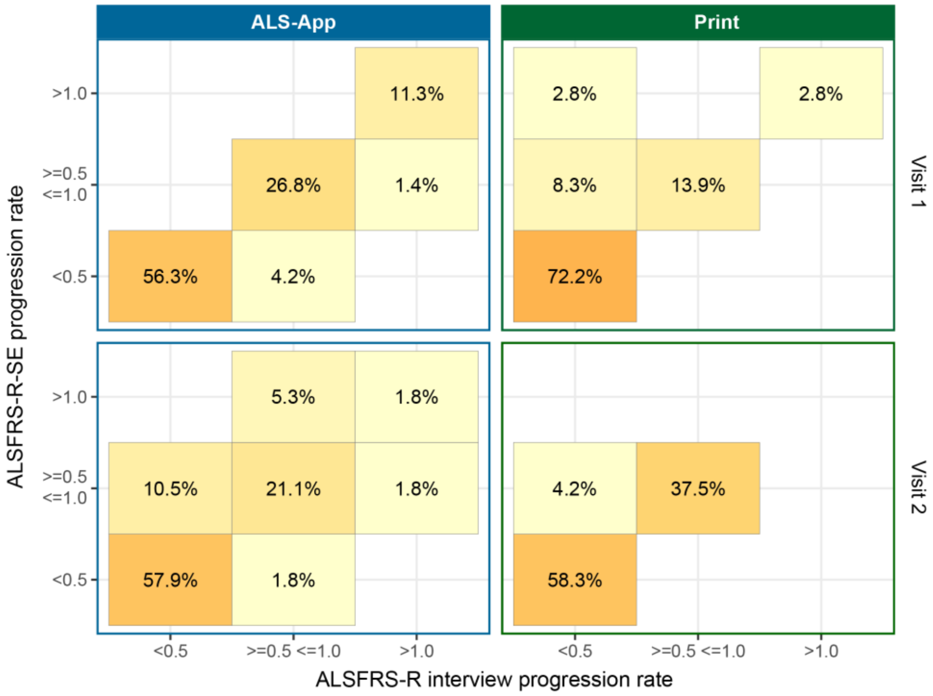

Supplement Figure S2: Comparison between progression rates resulting from the ALSFRS-R-SE and the harmonized ALSFRS-R interview. Progression rates were categorized as <0.5, ≥ 0.5 to ≤ 1.0, and greater than 1.0. The proportion of patients with the corresponding PR is shown in each field. A darker color indicates a higher proportion with a stronger correlation of PR, respectively. Fields on the bisecting axis indicate an exact match.
