# Supplementary material for: Agreement Between the Harmonized and the Self‐Explanatory Versions of the Revised ALS Functional Rating Scale in a Clinical Setting
Source: Muscle Nerve. 2025 Dec 2;73(2):250–9. doi: 10.1002/mus.70092 (PMC12803583; doi:10.1002/mus.70092)
Supplement: Supplementary file 3 — Table S1: mus70092‐sup‐0003‐Supplement_Table_S1.pdf. [file MUS-73-250-s005.pdf]

Supplement Table S1: Mean difference and percentage deviation upward and downward of individual ALSFRS-R items between the harmonized ALSFRS-R SOP, which was recorded as an interview, and the ALSFRS-R SE, which was completed by the patient at two time points. The SE was documented either in the ALS app or on a printed form.

	ALS App						Print					
	Visit 1			Visit 2			Visit 1			Visit 2		
**Item**	ΔM	Percent higher (95% CI)	Percent lower (95% CI)	ΔM	Percent higher (95% CI)	Percent lower (95% CI)	ΔM	Percent higher (95% CI)	Percent lower (95% CI)	ΔM	Percent higher (95% CI)	Percent lower (95% CI)
1	0.00	9.9 (4.1; 19.3)	9.9 (4.1; 19.3)	-0.09	5.3 (1.1; 14.6)	12.3 (5.1; 23.7)	0.06	11.1 (3.1; 26.1)	5.6 (0.7; 18.7)	0.04	12.5 (2.7; 32.4)	8.3 (1; 27)
2	-0.03	7 (2.3; 15.7)	8.5 (3.2; 17.5)	-0.16	7 (1.9; 17)	21.1 (11.4; 33.9)	-0.25	5.6 (0.7; 18.7)	22.2 (10.1; 39.2)	-0.08	8.3 (1; 27)	16.7 (4.7; 37.4)
3	0.06	12.7 (6; 22.7)	7 (2.3; 15.7)	0.02	10.5 (4; 21.5)	7 (1.9; 17)	-0.08	5.6 (0.7; 18.7)	13.9 (4.7; 29.5)	0.12	12.5 (2.7; 32.4)	0 (0; 14.2)
4	0.10	21.1 (12.3; 32.4)	15.5 (8; 26)	0.14	12.3 (5.1; 23.7)	5.3 (1.1; 14.6)	0.11	19.4 (8.2; 36	13.9 (4.7; 29.5)	0.08	20.8 (7.1; 42.2)	16.7 (4.7; 37.4)
5	-0.01	16.9 (9; 27.7)	9.9 (4.1; 19.3)	-0.05	8.8 (2.9; 19.3)	8.8 (2.9; 19.3)	-0.03	5.6 (0.7; 18.7)	8.3 (1.8; 22.5)	0.21	29.2 (12.6; 51.1)	8.3 (1; 27)
6	0.03	14.1 (7; 24.4)	12.7 (6; 22.7)	-0.02	8.8 (2.9; 19.3)	12.3 (5.1; 23.7)	-0.17	8.3 (1.8; 22.5)	22.2 (10.1; 39.2)	0.17	20.8 (7.1; 42.2)	4.2 (0.1; 21.1)
7	0.10	19.7 (11.2; 30.9)	11.3 (5; 21)	0.04	14 (6.3; 25.8)	10.5 (4; 21.5)	-0.03	11.1 (3.1; 26.1)	16.7 (6.4; 32.8)	0.04	12.5 (2.7; 32.4)	4.2 (0.1; 21.1)
8	0.10	18.3 (10.1; 29.3)	8.5 (3.2; 17.5)	0.11	15.8 (7.5; 27.9)	5.3 (1.1; 14.6)	-0.08	2.8 (0.1; 14.5)	8.3 (1.8; 22.5)	-0.04	12.5 (2.7; 32.4)	16.7 (4.7; 37.4)
9	0.03	21.1 (12.3; 32.4)	18.3 (10.1; 29.3)	0.09	19.3 (10; 31.9)	10.5 (4; 21.5)	0.03	16.7 (6.4; 32.8)	13.9 (4.7; 29.5)	0.21	25 (9.8; 46.7)	4.2 (0.1; 21.1)
10	0.08	18.3 (10.1; 29.3)	12.7 (6; 22.7)	-0.14	7 (1.9; 17)	12.3 (5.1; 23.7)	-0.11	19.4 (8.2; 36)	22.2 (10.1; 39.2)	-0.25	8.3 (1; 27)	20.8 (7.1; 42.2)
11	-0.03	11.3 (5; 21)	11.3 (5; 21)	-0.05	7 (1.9; 17)	12.3 (5.1; 23.7)	0.11	13.9 (4.7; 29.5)	2.8 (0.1; 14.5)	-0.04	12.5 (2.7; 32.4)	12.5 (2.7; 32.4)
12	0.07	5.6 (1.6; 13.8)	0 (0; 5.1)	0.04	3.5 (0.4; 12.1)	1.8 (0; 9.4)	0.08	8.3 (1.8; 22.5)	2.8 (0.1; 14.5)	0.04	8.3 (1; 27)	8.3 (1; 27)

ΔM = mean difference
