# Supplementary material for: Agreement Between the Harmonized and the Self‐Explanatory Versions of the Revised ALS Functional Rating Scale in a Clinical Setting
Source: Muscle Nerve. 2025 Dec 2;73(2):250–9. doi: 10.1002/mus.70092 (PMC12803583; doi:10.1002/mus.70092)
Supplement: Supplementary file 4 — Table S2: mus70092‐sup‐0004‐Supplement_Table_S2.pdf. [file MUS-73-250-s004.pdf]

Supplement Table S2: Mean progression rate (PR) in the individual cohorts and across visits. Categorization of PR was carried out in <0.5, ≥ 0.5 to ≤ 1.0, and greater than 1.0. The distribution of patients is reported in the categories.

	ALS App		Print	
**Characteristic**	**Visit 1**<br>N = 71	**Visit 2**<br>N = 57	**Visit 1**<br>N = 36	**Visit 2**<br>N = 24
**PR of ALSFRS-R interview**				
Mean (SD)	0.57 (0.58)	0.44 (0.31)	0.35 (0.44)	0.36 (0.27)
Median (Q1, Q3)	0.40 (0.24, 0.74)	0.39 (0.23, 0.62)	0.24 (0.10, 0.45)	0.28 (0.11, 0.59)
Min, Max	0.01, 3.00	0.01, 1.41	0.04, 2.61	0.06, 0.98
**PR of ALSFRS-R-SE**				
Mean (SD)	0.56 (0.62)	0.44 (0.32)	0.37 (0.48)	0.36 (0.27)
Median (Q1, Q3)	0.40 (0.21, 0.68)	0.37 (0.21, 0.62)	0.27 (0.11, 0.51)	0.27 (0.11, 0.61)
Min, Max	0.01, 3.70	0.01, 1.53	0.03, 2.79	0.05, 0.93
**PR categories of ALSFRS-R**				
PR category <0.5: n (%)	40 (56.34%)	39 (68.42%)	30 (83.33%)	15 (62.50%)
PR category ≥0.5 ≤1.0: n (%)	22 (30.99%)	16 (28.07%)	5 (13.89%)	9(37.50%)
PR category >1.0: n (%)	9 (12.68%)	2 (3.51%)	1 (2.78%)	0 (0.00%)
**PR categories of ALSFRS-R-SE**				
PR category <0.5: n (%)	43 (60.56%)	34 (59.65%)	26 (72.22%)	14 (58.33%)
PR category ≥0.5 ≤1.0: n (%)	20 (28.17%)	19 (33.33%)	8 (22.22%)	10 (41.67%)
PR category >1.0: n (%)	8 (11.27%)	4 (7.02%)	2 (5.56%)	0 (0.00%)
