# Supplementary material for: Agreement Between the Harmonized and the Self‐Explanatory Versions of the Revised ALS Functional Rating Scale in a Clinical Setting
Source: Muscle Nerve. 2025 Dec 2;73(2):250–9. doi: 10.1002/mus.70092 (PMC12803583; doi:10.1002/mus.70092)
Supplement: Supplementary file 5 — Table S3: mus70092‐sup‐0005‐Supplement_Table_S3.pdf. [file MUS-73-250-s002.pdf]

Supplement Table S3: Agreement and correlation of progression rates between the harmonized ALSFRS-R SOP recorded as an interview and the ALSFRS-R-SE completed by the patient at two timepoints.

Kohort	Visit	Percent Agreement (95% CI)	Kendalls τ	Spearman	p-value[1]
ALS App	1	94.4 (86.2; 98.4)	0.93*	0.94	0.135
	2	80.7 (68.1; 90.0)	0.74*	0.77*	0.102
Print	1	88.9 (73.9; 96.9)	0.7*	0.71*	0.135
	2	95.8 (78.9; 99.9)	0.92*	0.92*	0.607

* p < 0.001; 1 Stuart-Maxwell-Test
